# Sources of polycyclic aromatic hydrocarbons exposure and their effects on glycolipid metabolism in pregnant women and their newborn in Haikou City, China

**DOI:** 10.3389/fpubh.2024.1510517

**Published:** 2025-01-20

**Authors:** Xiaomei Cheng, Haifeng Gao, Qiaojun Li, Naifan Zhang, Ying Lu

**Affiliations:** ^1^School of Public Health, Hainan Academy of Medical Sciences, Hainan Medical University, Hainan, China; ^2^School of Public Health, Xinjiang Medical University, Xinjiang, China

**Keywords:** glycolipid metabolism, Hainan, new born, polycyclic aromatic hydrocarbon, pregnancy

## Abstract

**Background:**

Polycyclic aromatic hydrocarbons (PAHs) are a class of large organic compounds that persist in the environment and are classified as an important subset of persistent organic pollutants (POPs). This study aimed to assess PAH exposure in pregnant women and newborns in Haikou City, China, and evaluate their impact on glycolipid metabolism.

**Methods:**

A total of 300 pregnant women and their newborns were selected for the study between May 2022 and December 2023. Data on lifestyle and dietary habits were collected, and PAH levels in venous blood and umbilical cord blood were measured using gas chromatography-mass spectrometry. Glycolipid metabolism indicators, including fasting plasma glucose (FPG), triglycerides (TG), total cholesterol (TC), high-density lipoprotein (HDL), and low-density lipoprotein (LDL), were also measured. Correlation and regression analyses were conducted to explore the relationships between PAH exposure and metabolic indicators.

**Results:**

Thirteen PAHs were detected in both maternal and cord blood. The median concentrations of ΣPAHs (the total concentration of the 16 detected PAHs) were 11.211 μg/L in maternal blood and 10.921 μg/L in newborns. Significant correlations were observed between PAH exposure and glycolipid metabolism, with PAH exposure linked to reduced levels of TC and TG. Factors such as proximity to roads, cooking fuel type, and dietary habits influenced PAH levels. Higher education levels were associated with lower PAH concentrations, whereas living near roads and using gas as cooking fuel increased exposure.

**Conclusion:**

There is a notable risk of PAH exposure in pregnant women and newborns in Haikou, influenced by environmental and lifestyle factors. PAH exposure during pregnancy may affect glycolipid metabolism in both mothers and newborns, highlighting the need for interventions to reduce exposure.

## Introduction

Polycyclic aromatic hydrocarbons (PAHs) are a class of large organic compounds composed of 2–7 fused aromatic rings. They are primarily formed through the incomplete combustion of fossil fuels, biomass, and other organic materials. PAHs have garnered significant attention due to their persistence in the environment and their classification as a critical subset of persistent organic pollutants [POPs; (1, 2)]: the compounds are highly stable in the environment, and their hydrophobic nature enables them to persist in soils, sediments, and water bodies. They can also bind to atmospheric particulate matter, facilitating their long-range atmospheric transport (LRAT) across national borders. This phenomenon has been observed in remote regions such as the Arctic and Antarctic (3), where PAHs have accumulated, serving as “cold reservoirs” for POPs (4, 5). The U.S. Environmental Protection Agency (EPA) has identified 16 PAHs as priority pollutants, highlighting their potential health risks and the need for regulatory control (6). Their widespread distribution, combined with their tendency to bioaccumulate, makes PAHs a significant global public health concern.

Human exposure to PAHs occurs through various routes, including inhalation of polluted air, ingestion of contaminated food or water, and dermal contact with PAH-containing materials. As a result, PAHs are ubiquitous in human organs and have been detected in the urine, blood, and hair of the general population (7, 8). This widespread exposure is primarily attributed to the presence of PAHs in traffic-related air pollution, industrial emissions, and the combustion of biomass and fossil fuels. For individuals living in urban environments or near industrial zones, exposure is often unavoidable, resulting in both chronic and acute health risks, including respiratory disorders, cardiovascular diseases, and immunosuppression (9). Moreover, certain PAHs, such as benzo[a]pyrene, are recognized carcinogens, and long-term exposure is associated with an increased risk of developing cancers, particularly lung, skin, and bladder cancer.

The endocrine-disrupting potential of PAHs is also a critical concern for public health systems. As members of a class of chemicals known as endocrine-disrupting chemicals (EDCs), PAHs can interfere with the body's hormonal system by binding to hormone receptors or altering hormone production and metabolism. This disruption can result in a wide range of health issues, including reproductive harm, developmental disorders, metabolic diseases, and cancer (10). Moreover, PAH exposure may indirectly affect blood lipid levels through intermediate mechanisms such as inflammation and oxidative stress (11). For example, environmental PAHs have been shown to exert toxic effects on nuclear receptor signaling, disrupt endogenous metabolism, and induce cellular stress (12). PAHs are particularly concerning because they can cross the placental barrier, exposing fetuses to these chemicals during critical windows of development. Prenatal exposure to PAHs has been linked to various developmental issues, including lower birth weight, reduced head circumference, and impaired neurological function (13, 14). These findings underscore the importance of studying PAH exposure during pregnancy and its potential long-term effects on both maternal and fetal health.

Pregnant women are particularly susceptible to environmental pollutants due to the physiological changes that occur during pregnancy (13, 14). Increased metabolic activity and blood flow during gestation can enhance the absorption and distribution of PAHs within the body. Additionally, hormonal fluctuations during pregnancy may influence the metabolism of PAHs, potentially increasing their bioactivation and toxicity. Consequently, pregnant women exposed to PAHs may experience disruptions in metabolic processes, particularly glycolipid metabolism, which regulates blood sugar and lipids—processes essential for maintaining energy balance and overall health. Disruptions in glycolipid metabolism during pregnancy can lead to complications such as gestational diabetes and dyslipidemia, which, in turn, elevate the risk of adverse pregnancy outcomes (13, 14).

Hainan Island, located at the southernmost tip of China, offers a unique environment for studying the effects of PAHs on human health. With its tropical climate and rapid urbanization, the island faces significant environmental pollution challenges, particularly in its capital city, Haikou—a major shipping hub and economic center—which has led to increased emissions from industrial activities and traffic. Despite being surrounded by the sea and generally having excellent air quality, PAHs were detected in atmospheric PM2.5 samples collected in Haikou, with the exception of fluorene (Flu). Among the detected compounds, four-ring PAHs accounted for the highest proportion (15). PAHs are commonly found in marine environments. High molecular weight PAHs (also known as high-ring PAHs, containing 4–7 benzene rings) tend to adhere to particle surfaces and settle into sediments, whereas low molecular weight PAHs (low-ring PAHs, containing 2–3 benzene rings) are absorbed by aquatic organisms through various exposure pathways, including inhalation, skin contact, and ingestion. These compounds can cause both acute and chronic toxic effects (16). In Hainan's Yangpu Bay and Haikou Bay, low molecular weight PAHs, such as naphthalene, fluorene, phenanthrene (Phe), and acenaphthene, primarily derived from petroleum hydrocarbon contamination, dominate in seawater and sediments (17, 18). PAHs have been detected in various marine products from Hainan, including fish, shrimp, crabs, and shellfish. Among these, three-ring PAHs are the most prevalent, accounting for 41.58% to 83.35% (19, 20). Among the 16 PAHs detected, phenanthrene and naphthalene are the most abundant in coral tissue. PAHs in aquatic organisms can enter the human body through the food chain. Additionally, remote transfer via atmospheric currents and sea-air transport contributes to the widespread exposure to PAHs in Hainan. Studies have demonstrated that PAH levels in pregnant women and newborns are primarily influenced by factors such as the distance between their residences and roads, indoor ventilation conditions, and cooking methods during pregnancy. Similar findings have been observed in other inland cities in China, such as Urumqi in Xinjiang, further confirming that traffic emissions are a significant source of human exposure to PAHs. Haikou, the capital of Hainan Province and a key water and land transportation hub, has been found to contain PAHs in nearby water bodies and aquatic organisms. However, whether the PAHs detected in humans are directly related to those in the aquatic environment and organisms remains to be further investigated.

In this study, we aimed to provide valuable insights into the exposure of pregnant women and newborns in Haikou to PAHs, focusing on their internal PAH burden and the effects of PAHs on glycolipid metabolism. By assessing PAH levels in maternal and neonatal blood samples, the study seeks to identify sources of PAH exposure in subtropical island climates and evaluate their potential impact on maternal and fetal health. Understanding these relationships is essential for developing strategies to reduce PAH exposure and mitigate its harmful effects on vulnerable populations, particularly pregnant women and their offspring.

## Methods

### Study subjects

A total of 300 pairs of pregnant women and their newborns, admitted to Haikou Hospital affiliated with the Xiangya School of Medicine at Central South University between May 2022 and December 2023, were included in this study. According to the 2018 statistical yearbook, there were 134,700 newborns in Hainan Province. Haikou City, with a permanent resident population of 2.3023 million (accounting for 24.6% of Hainan Province's total population of 9.3432 million), was estimated to have ~33,000 newborns that year. In a preliminary study of non-occupationally exposed women, the concentration of 1-hydroxypyrene was 0.042 ± 0.4 μg/mmol Cr. Using this data, the sample size for the current study was determined based on the formula for cross-sectional surveys, calculated with PASS software, to be 300.

The inclusion criteria was as followings: (1) Pregnant women aged 18 years or older. (2) Permanent residents of Haikou (residing for more than 1 year). (3) No history of smoking or alcohol consumption. (4) Consent to provide relevant biological samples for the study and willingness to cooperate with researchers, including providing personal contact information. (5) Singleton full-term delivery. Participants were excluded if they met any of the following criteria: (1) History of acute or chronic illnesses such as hypertension, diabetes, or coronary heart disease. (2) Diagnosis of gestational diabetes or gestational hypertension during pregnancy. (3) History of hereditary or infectious diseases. (4) History of stillbirth, miscarriage, or medication use.

Data collected from all participants included age, ethnicity, health status, family history, reproductive history, household fuel use, heating methods, and dietary habits. Biological samples were obtained, including peripheral venous blood from the mothers and umbilical cord blood from the newborns. The study was conducted with the approval of the Ethics Committee of Hainan Academy of Medical Sciences (HYLL-2021-108). All participants provided written informed consent, and all procedures adhered to the principles outlined in the Declaration of Helsinki.

### Blood sample collection and processing

Peripheral venous blood (10 mL) from pregnant women and umbilical cord blood (10 mL) from newborns were collected by a professional nurse using EDTA anticoagulation tubes. The samples were centrifuged within 30 min of collection at 3,500 rpm for 5 min, and the supernatant (plasma) was transferred to cryovials using a micropipette and stored at −80°C.

### Detection of PAH levels

A mixed standard solution of 16 PAHs (ultra-pure, provided by Aladdin, Tianjin, China) and a gas chromatography-tandem triple quadrupole mass spectrometer (TSQ9000, Thermo Fisher Scientific, USA) were used to detect PAH levels in maternal venous blood and newborn umbilical cord blood through gas chromatography-mass spectrometry (GC-MS). Serum samples were thawed at room temperature before analysis, and 4 mL of a 0.4 mol/L sodium hydroxide ethanol-water solution was prepared for the procedure. In brief, 500 μL of serum was mixed with 4 mL of sodium hydroxide ethanol-water solution and incubated in a water bath at 60°C for 30 min. Next, 3 mL of n-hexane was added for liquid-liquid extraction, and the organic layer was concentrated to 0.5 mL using a nitrogen blow-down apparatus. The residue was then reconstituted to 1 mL with n-hexane and transferred to sample vials for GC-MS analysis. If the concentration of a detected compound was below the minimum detection limit, it was recorded as 0. Detailed GC-MS parameters for the 16 PAHs are provided in Supplementary Table 1.

The quality control of the detection was performed as followings: before each GC-MS analysis, automatic tuning of the mass spectrometer (MS) was performed. The GC and MS were then set to the operating conditions specified by the analytical method and placed in standby mode. A 1.0 μL sample of n-hexane was injected directly into the GC injection port, and the analytical method was run until the n-hexane peak appeared. The ion abundance of its mass fragments was required to meet specified criteria, with a stable baseline and no extraneous peaks. If these conditions were not met, the ion source of the mass spectrometer was cleaned. For each batch of samples (up to 20), a blank experiment was conducted. The concentration of target compounds in the blank results was required to be below the method detection limit. If it exceeded the limit, the reagent blank, instrument system, and pretreatment process were inspected. Each batch of samples (up to 20) included one pair of parallel samples. The relative deviation between the parallel sample measurements was required to be <30%.

### Measurement of 8-OHdG levels

An enzyme-linked immunosorbent assay (ELISA) was used to measure the levels of 8-OHdG in maternal plasma and newborn umbilical cord blood serum. The process followed the manufacturer's instructions for the ELISA kit: briefly, the samples were thawed at 4°C for 30 min and centrifuged at 3,000 rpm and 4°C for 5 min, retaining the supernatant. For standard wells, 50 μL of standards at different concentrations were used, while 40 μL of sample diluent and 10 μL of the sample were added to the sample wells. The plate was incubated at 37°C for 60 min. A 20-fold diluted washing solution was used to wash the wells five times, with 350 μL added per wash and a 30-s standing period before discarding. Afterward, 50 μL each of Color Reagents A and B were added, incubated at 37°C for 15 min, and the reaction was stopped with 50 μL of Stop Solution. A standard curve was plotted using OD values and standard concentrations, and sample concentrations were calculated by substituting measured OD values into the regression equation, with final concentrations adjusted by the dilution factor.

### Measurement of glucose and lipid metabolism

Fasting blood glucose (FBG) concentrations were measured using a glucose oxidase ELISA kit, while triglycerides (TG), total cholesterol (TC), high-density lipoprotein (HDL), and low-density lipoprotein (LDL) levels in maternal plasma were analyzed with a biochemical analyzer (Mindray BS-380, China).

Gestational diabetes was diagnosed based on the International Association of Diabetes and Pregnancy Study Groups (IADPSG) criteria, with FBG levels ≥5.1 mmol/L (21). Dyslipidemia was defined according to the 2016 Chinese guidelines for the prevention and treatment of adult dyslipidemia, with normal ranges as follows: TG < 2.26 mmol/L, TC < 6.22 mmol/L, LDL < 4.14 mmol/L, and HDL ≥ 1.04 mmol/L.

### Statistical analysis

Data entry was conducted using Epidata 3.1 with double-entry verification, and statistical analysis was performed with SPSS 25.0. Quantitative data were expressed as mean ± standard deviation and compared using *t*-tests, while categorical data were analyzed using chi-square tests. Spearman correlation analysis examined associations between variables such as PAH and 8-OHdG levels, and multiple linear regression identified influencing factors. Statistical significance was defined as α = 0.05.

## Results

### General conditions and pregnancy lifestyle of pregnant women in Haikou city

The study included 300 pairs of pregnant women and their newborns. The age of the pregnant women ranged from 18 to 44 years, with a mean age of 31 years. Most participants (94.0%) were of Han ethnicity, while 6.0% belonged to minority groups. Regarding education, 7.9% had attained a college degree or higher (Table 1). The majority of women lived in homes that had not been recently renovated, used gas or natural gas for cooking, and reported good ventilation. A significant proportion resided near roads, though few lived close to industrial sites or waste treatment facilities. In terms of dietary habits, most consumed fruits and vegetables daily, while seafood and meat were consumed less frequently. Fried or grilled foods were largely avoided, and nearly all participants drank tap water. Detailed information on lifestyle factors is provided in Tables 2, 3.

**Table 1:** General demographic characteristics and assignments of pregnant women in Haikou (*n* = 300).

**Characteristic**	**Classification**	**Assignment**	**Number of cases**	**Proportion (%)**
Age (years)	18	Continuous variable	55	18.3
	27	**–**	84	28.0
	31	**–**	84	28.0
	35–44	**–**	77	25.7
Education^a^	Junior high school or below	1	88	30.4
	High school	2	179	61.7
	College/Undergraduate	3	18	6.2
	Graduate school	4	5	1.7
Ethnicity	Han	1	282	94.0
	Others	2	18	6.0

^a^Missing 10 cases.

**Table 2:** Living and environmental conditions during pregnancy in Haikou pregnant women (*n* = 300).

**Characteristic**	**Classification**	**Assignment**	**Number of cases**	**Proportion (%)**
Cooking method during pregnancy^a^	Induction cooker	1	39	14.2
	Gas	2	128	46.5
	Natural gas	3	108	39.3
Home renovation during pregnancy^b^	No	0	263	94.6
	Yes	1	15	5.4
Distance from home to road^c^	Far (≥800 m)	1	60	20.6
	Close (400–800 m)	2	169	58.1
	Beside the road (≤ 400 m)	3	62	21.3
Daily time spent on the road^d^	Less than 1 h	1	191	69.2
	1–2 h	2	70	25.4
	More than 2 h	3	15	5.4
Presence of large waste processing facility near residence^e^	No	0	275	98.9
	Yes	1	3	1.1
Presence of chemical plants, refineries, etc. near residence^f^	No	0	268	97.1
	Yes	1	8	2.9
Ventilation condition of residence during pregnancy^g^	Very good	1	183	66.3
	Good	2	92	33.3
	Poor	3	1	0.4
Presence of carpet or rug in the residence^h^	No	0	249	90.2
	Yes	1	27	9.8

^a^Missing 25 cases; ^b^Missing 10 cases; ^c^Missing 9 cases; ^d^Missing 24 cases; ^e^Missing 22 cases; ^f^Missing 24 cases; ^g^Missing 24 cases; ^h^Missing 24 cases.

**Table 3:** Dietary conditions during pregnancy in Haikou pregnant women (*n* = 300).

**Characteristic**	**Classification**	**Assignment**	**Number of cases**	**Proportion (%)**
Seafood consumption during pregnancy^a^	Every day	3	14	5.1
	Frequently	2	87	31.8
	Rarely	1	155	56.6
	None	0	18	6.6
Meat consumption during pregnancy^b^	Every day	3	121	44.0
	Frequently	2	117	42.5
	Rarely	1	36	13.1
	None	0	1	0.4
Bean and dairy products consumption^c^	Every day	3	57	20.8
	Frequently	2	138	50.4
	Rarely	1	75	27.4
	None	0	4	1.4
Vegetable and fruit consumption^d^	Every day	3	186	67.9
	Frequently	2	77	28.1
	Rarely	1	10	3.6
	None	0	1	0.4
Egg consumption^e^	Every day	3	136	51.1
	Frequently	2	5	1.9
	Rarely	1	17	6.4
	None	0	108	40.6
Fried or grilled food consumption^f^	Every day	3	2	0.7
	Frequently	2	17	6.2
	Rarely	1	6	2.2
	None	0	250	90.9
Water source during pregnancy	Well water	1	7	2.3
	Tap water	2	276	92.0
	Mineral water	3	17	5.7
Tea drinking habit during pregnancy^g^	No	0	235	84.5
	Yes	1	43	15.5

^a^Missing 26 cases; ^b^Missing 25 cases; ^c^Missing 26 cases; ^d^Missing 26 cases; ^e^Missing 34 cases; ^f^Missing 25 cases; ^g^Missing 22 cases.

### PAH levels in pregnant women's venous blood and newborn cord blood

Among the 16 types of PAHs initially analyzed, 13 were detected in both maternal and newborn samples. Certain PAHs, including anthracene (Ant) and phenanthrene (Phe) were present in 100% of maternal blood samples (Table 4). In contrast, some PAHs, such as dibenz[a,h]anthracene (DahA), had much lower detection rates, with only 31% of maternal samples testing positive (Table 4). A similar detection profile was observed in newborn cord blood, where PAHs like anthracene and phenanthrene were detected in all samples, whereas others, such as dibenz[a,h]anthracene, were found in only 19.7% of cases (Table 4). Phenanthrene exhibited the highest median concentration among the detected PAHs, with levels of 1.837 μg/L in maternal venous blood and 1.768 μg/L in newborn cord blood (Table 4). In contrast, dibenz[a,h]anthracene showed the lowest concentrations, often being undetectable or present only at trace levels (Table 4). Other PAHs, such as benzo[a]pyrene (BaP) and benzo[b]fluoranthene (BbFA), demonstrated high detection rates, approaching 99.0% in both maternal and cord blood samples, highlighting widespread exposure among the study population (Table 4).

**Table 4:** PAH levels in venous blood of pregnant women and umbilical cord blood of newborns in Haikou and related correlation coefficients (rs; *n* = 300).

**PAHs**	**Venous blood**			**Umbilical cord blood**		
	**Detection rate [*****n*** **(%)]**	**Concentration M (P25, P75;** μ**g/L)**	**Extreme value (**μ**g/L)**	**Detection rate (%)**	**Concentration M (P25, P75;** μ**g/L)**	**Extreme value (**μ**g/L)**
Acenaphthylene (Ace)	Not detected	–	–	Not detected	–	–
Acenaphthene (Acy)	284 (94.7%)	0.523 (0.460, 0.677)	0.000–0.088	298 (99.3%)	0.634 (0.474, 0.659)	0.000–0.855
Anthracene (Ant)	300 (100.0%)	1.812 (1.760, 1.910)	1.521–2.516	300 (100.0%)	1.751 (1.717, 1.801)	1.522–2.654
Benzo[a]anthracene (BaA)	281 (93.7%)	0.541 (0.529, 0.661)	0.000–0.733	297 (99.0%)	0.672 (0.533, 0.680)	0.000–0.816
Benzo[a]pyrene (BaP)	280 (93.3%)	0.687 (0.677, 0.701)	0.000–0.835	297 (99.0%)	0.701 (0.679, 0.722)	0.000–0.849
Benzo[b]fluoranthene (BbFA)	289 (96.3%)	0.618 (0.611, 0.631)	0.000–0.766	299 (99.7%)	0.632 (0.611, 0.654)	0.000–0.778
Benzo[ghi]perylene (BghiP)	Not detected	–	–	Not detected	–	–
Benzo[k]fluoranthene (BkFA)	293 (97.7%)	0.617 (0.611, 0.630)	0.000–0.766	299 (99.7%)	0.631 (0.611, 0.654)	0.000–0.778
Chrysene (Chr)	279 (93.0%)	0.550 (0.539, 0.667)	0.000–0.741	297 (99.0%)	0.679 (0.543, 0.688)	0.000–0.823
Dibenzo[ah]anthracene (DahA)	93 (31.0%)	0.000 (0.000, 2.432)	0.000–2.593	59 (19.7%)	0 (0, 0)	0.000–2.544
Fluoranthene (FLa)	298 (99.3%)	1.520 (1.500, 1.549)	0.000–2.048	299 (99.7%)	1.530 (1.504, 1.552)	0.000–2.187
Fluorene (FLu)	Not detected	–	–	Not detected	–	–
Indeno[123-cd]pyrene (InP)	100 (33.3%)	0.000 (0.000, 1.695)	0.000–1.991	92 (30.7%)	0.000 (0.000, 1.690)	0.000–1.792
Naphthalene (Nap)	240 (80.0%)	0.463 (0.214, 0.700)	0.000–4.077	289 (96.3%)	0.412 (0.279, 0.467)	0.000–2.503
Phenanthrene (Phe)	300 (100.0%)	1.837 (1.779, 1.926)	1.538–2.539	300 (100.0%)	1.768 (1.733, 1.819)	1.538–2.046
Pyrene (Pyr)	298 (99.3%)	1.592 (1.571, 1.625)	0.000–2.119	299 (99.7%)	1.606 (1.580, 1.628)	0.000–2.258
ΣPAHs	–	11.211 (10.700, 14.619)	3.822–17.954	–	10.921 (10.698, 12.734)	3.261–16.717

**Figure 1:**
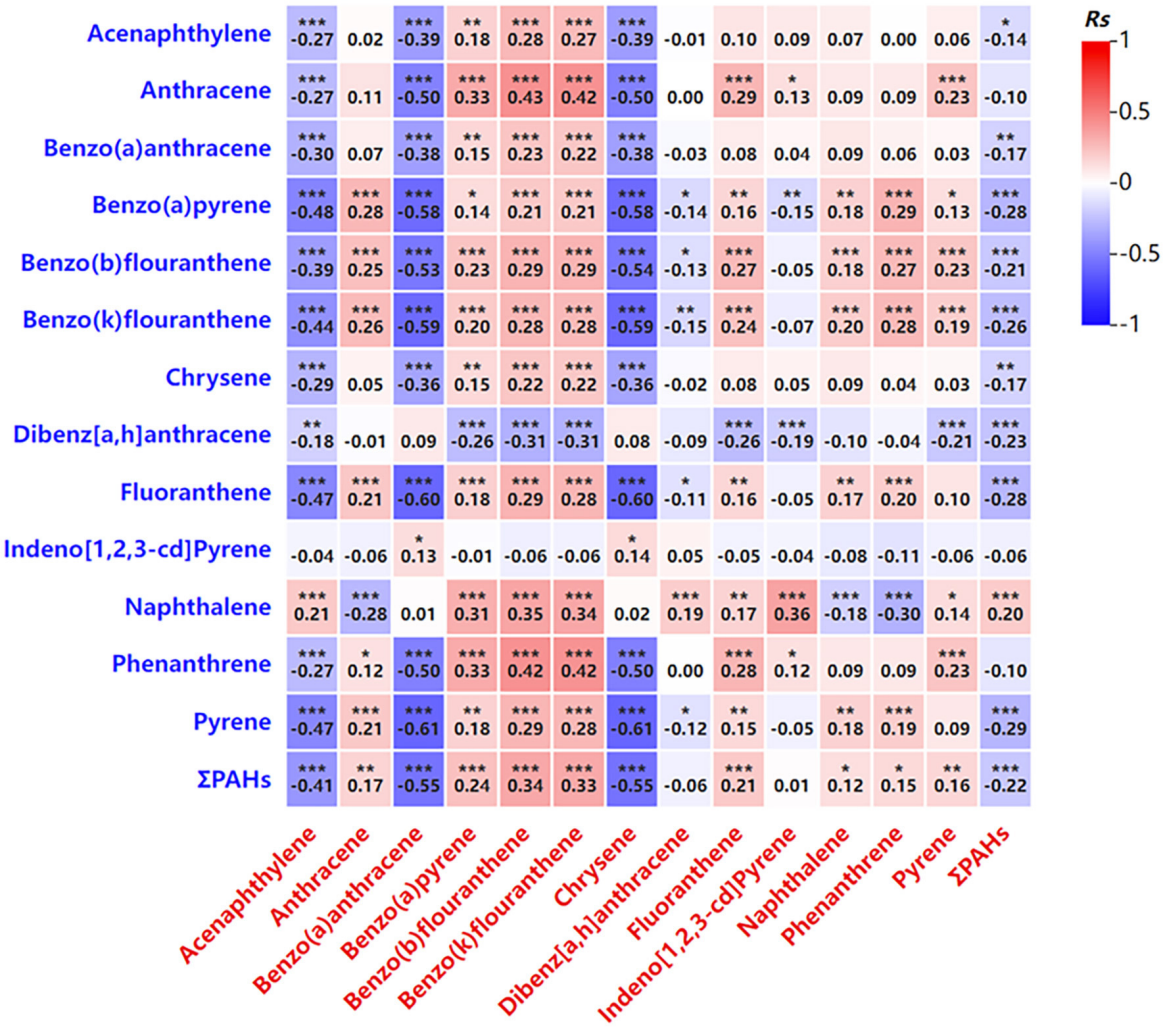
Correlation analysis of the PAH levels in venous blood and in umbilical cord blood. Blue represents PAH level in venous blood. Red represents PAH levels in umbilical cord blood. *P < 0.05, **P < 0.01, ***P < 0.001.

The total concentration of PAHs (ΣPAHs, the total concentration of the 16 PAHs tested) in maternal blood was positively correlated with the concentrations of specific PAHs in newborn cord blood, including benzo[a]pyrene, benzo[b]fluoranthene, and benzo[k]fluoranthene (Table 4; Figure 1). Conversely, a negative correlation was observed between maternal PAH concentrations and certain PAHs in cord blood, such as chrysene (Chr) and benzo[a]anthracene (BaA; Table 4; Figure 1). These findings indicate a strong relationship between maternal PAH exposure during pregnancy and newborn exposure, likely due to the transplacental transfer of these compounds.

The analysis revealed that certain PAHs in maternal blood, such as acenaphthylene and benzo[a]anthracene, were negatively correlated with their concentrations in newborn cord blood, while others, including benzo[a]pyrene, exhibited a positive correlation (Table 4; Figure 1). These findings suggest a complex interaction between maternal PAH exposure and placental transfer mechanisms, which influence the distribution of these compounds in the fetus.

### Factors influencing PAH concentration in pregnant women's blood

Several factors were identified as influencing PAH concentrations in maternal blood, including time spent near roads, the presence of carpets in the home, and the mothers' educational background (Table 5). Higher PAH concentrations were associated with more time spent near roads and the presence of household carpets. In contrast, higher educational attainment was linked to lower PAH levels. Additionally, increased consumption of fruits and vegetables during pregnancy was correlated with reduced maternal PAH concentrations. Detailed information on these influencing factors is provided in Table 5.

**Table 5:** Multiple linear regression analysis of PAH concentrations in venous blood of pregnant women in Haikou (*n* = 300).

**PAHs Type**	**Variable**	**β**	**SD**	***t*-Value**	***P*-Value**
ΣPAHs	Intercept	7.596	4.512	1.684	0.094
	(Daily time on the road during pregnancy)	1.404	0.607	2.313	0.022
Acenaphthylene (Acy)	Intercept	0.225	0.189	1.192	0.235
	(Carpet or rug in the house)	0.106	0.048	2.177	0.030
Benzo[a]pyrene (BaP)	Intercept	0.508	0.274	1.852	0.065
	(Carpet or rug in the house)	0.156	0.071	2.211	0.028
Benzo[b]fluoranthene (BbFA)	Intercept	0.576	0.246	2.343	0.020
	X2 (Education)	−0.070	0.031	−2.225	0.027
	(Carpet or rug in the house)	0.138	0.063	2.183	0.030
Benzo[k]fluoranthene (BkFA)	Intercept	0.534	0.246	2.169	0.031
	(Distance from home to road)	−0.063	0.030	−2.098	0.037
	(Carpet or rug in the house)	0.135	0.063	2.130	0.034
Dibenzo[ah]anthracene (DahA)	Intercept	0.075	0.589	0.128	0.899
	(Daily time on the road during pregnancy)	0.273	0.079	3.448	0.001
	(Ventilation condition of the house)	−0.230	0.093	−2.484	0.014
Indeno[123-cd]pyrene (InP)	Intercept	0.141	0.424	0.332	0.740
	(Daily time on the road during pregnancy)	0.165	0.057	2.902	0.004
	(Ventilation condition of the house)	−0.159	0.067	−2.391	0.018
Naphthalene (Nap)	Intercept	0.287	0.185	1.555	0.121
	(Cooking method during pregnancy)	0.046	0.021	2.175	0.031
	(Vegetable and fruit consumption)	−0.065	0.026	−2.541	0.013

### 8-OHdG metabolism levels and their correlation with PAH levels

The median 8-OHdG level in maternal blood was 56.5 ng/mL, while newborn cord blood had a median of 54.0 ng/mL, indicating oxidative stress in both groups. No significant correlation was found between PAH concentrations and 8-OHdG levels in maternal blood. However, in newborn umbilical cord blood, significant positive correlations were observed between several PAHs—including dibenz[a,h]anthracene (DahA), fluoranthene (FLa), indeno[1,2,3-cd]pyrene (InP), and pyrene (Pyr)—and 8-OHdG levels (Table 6). This suggests that PAH exposure during pregnancy contributes to oxidative stress in newborns. This heightened fetal vulnerability highlights the importance of minimizing PAH exposure during pregnancy to protect against oxidative damage and its potential long-term health consequences for the child.

**Table 6:** Correlation between PAHs exposure levels in venous blood of pregnant women and umbilical cord blood of newborns in Haikou and 8-OHdG levels (*n* = 300).

**PAHs**	**8-OHdG^a^ (Venous blood)**		**8-OHdG^b^ (Umbilical cord blood)**	
	**rs**	***P***	**rs**	***P***
Acenaphthylene (Ace)	–	–	–	–
Acenaphthene (Acy)	−0.106	0.198	0.082	0.320
Anthracene (Ant)	−0.024	0.771	−0.047	0.567
Benzo[a]anthracene (BaA)	0.012	0.888	0.126	0.124
Benzo[a]pyrene (BaP)	0.157	0.055	0.121	0.139
Benzo[b]fluoranthene (BbFA)	0.144	0.078	0.141	0.086
Benzo[ghi]perylene (BghiP)	–	–	–	–
Benzo[k]fluoranthene (BkFA)	0.097	0.236	0.134	0.102
Chrysene (Chr)	−0.001	0.992	0.128	0.118
Dibenzo[ah]anthracene (DahA)	0.018	0.825	0.223	0.006
Fluoranthene (FLa)	0.042	0.608	0.194	0.018
Fluorene (FLu)	–	–	–	–
Indeno[123-cd]pyrene (InP)	0.044	0.590	0.190	0.020
Naphthalene (Nap)	−0.148	0.071	−0.118	0.150
Phenanthrene (Phe)	−0.030	0.718	−0.120	0.145
Pyrene (Pyr)	0.016	0.850	0.187	0.022
ΣPAHs	−0.014	0.862	0.177	0.030

^a^Correlation with PAHs concentration in venous blood; ^b^Correlation with PAHs concentration in umbilical cord blood.

### Blood glucose and lipid metabolism in pregnant women and newborns

As shown in Table 7, triglyceride (TG) levels in maternal blood were negatively correlated with those in newborn blood, suggesting a potential inverse relationship in metabolic function between mothers and their newborns. Other metabolic markers, including total cholesterol, fasting blood glucose, and HDL cholesterol, were also analyzed, further confirming the negative association between TG levels in maternal and newborn blood (Table 7).

**Table 7:** Glucose and lipid metabolism levels in venous blood of pregnant women and umbilical cord blood of newborns in Haikou and related correlation coefficients (rs; *n* = 300).

	**Venous blood**	**Umbilical cord blood**	**rs**	***P***
FPG (mmol/L)	4.215 (3.383, 5.108)	3.700 (2.590, 5.055)	0.079	0.186
TC (mmol/L)	6.600 (5.245, 8.340)	1.665 (1.240, 2.243)	−0.085	0.154
TG (mmol/L)	3.530 (2.763, 4.488)	0.400 (0.320, 0.530)	−0.121	0.041
HDL (mmol/L)	1.825 (1.480, 2.258)	0.880 (0.670, 1.198)	−0.060	0.315
LDL (mmol/L)	2.980 (2.280, 3.790)	0.460 (0.330, 0.630)	−0.076	0.203

### Correlation between PAH and 8-OHdG levels and metabolic levels

**Figure 2:**
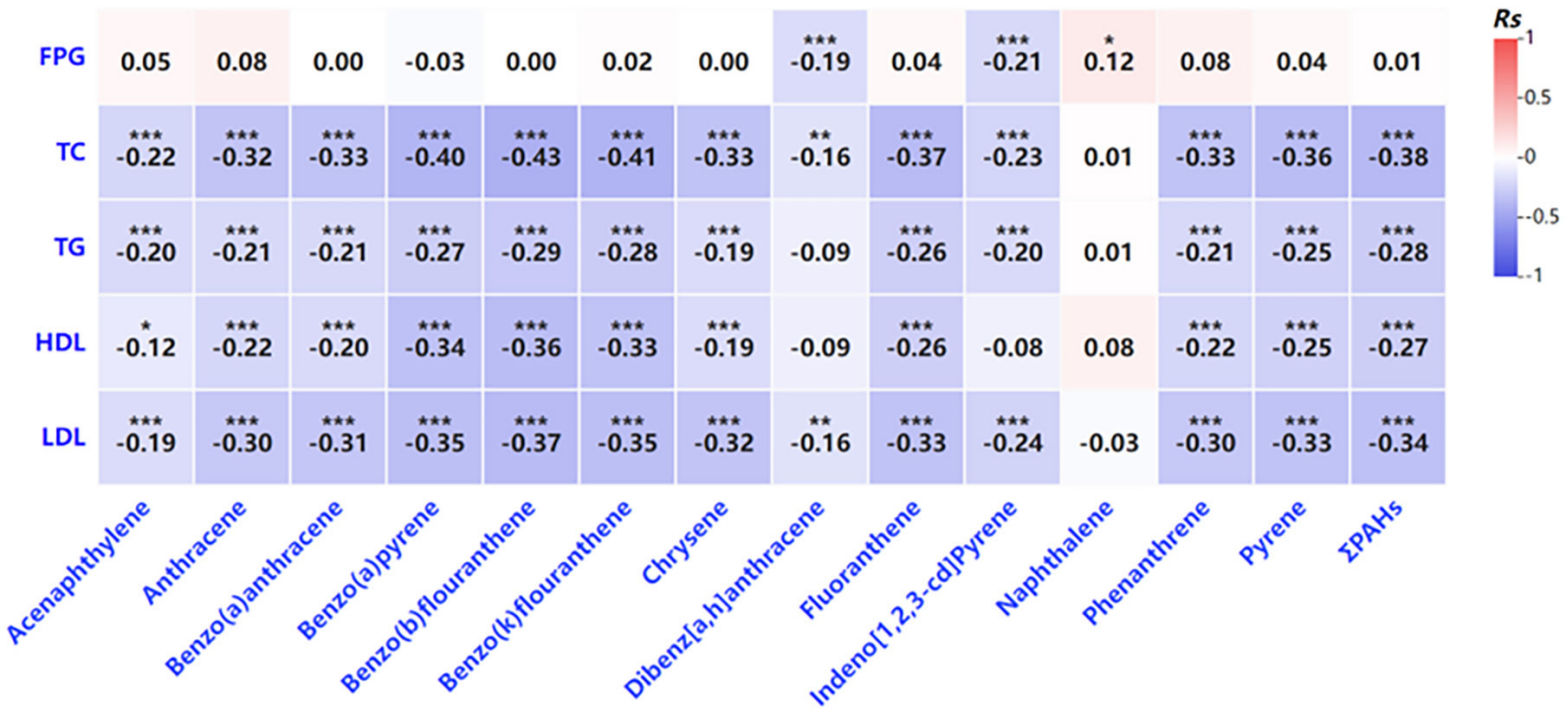
Correlation analysis of the indicators related to glycolipid metabolism PAH levels in venous blood. *P < 0.05, **P < 0.01, ***P < 0.001.

**Figure 3:**
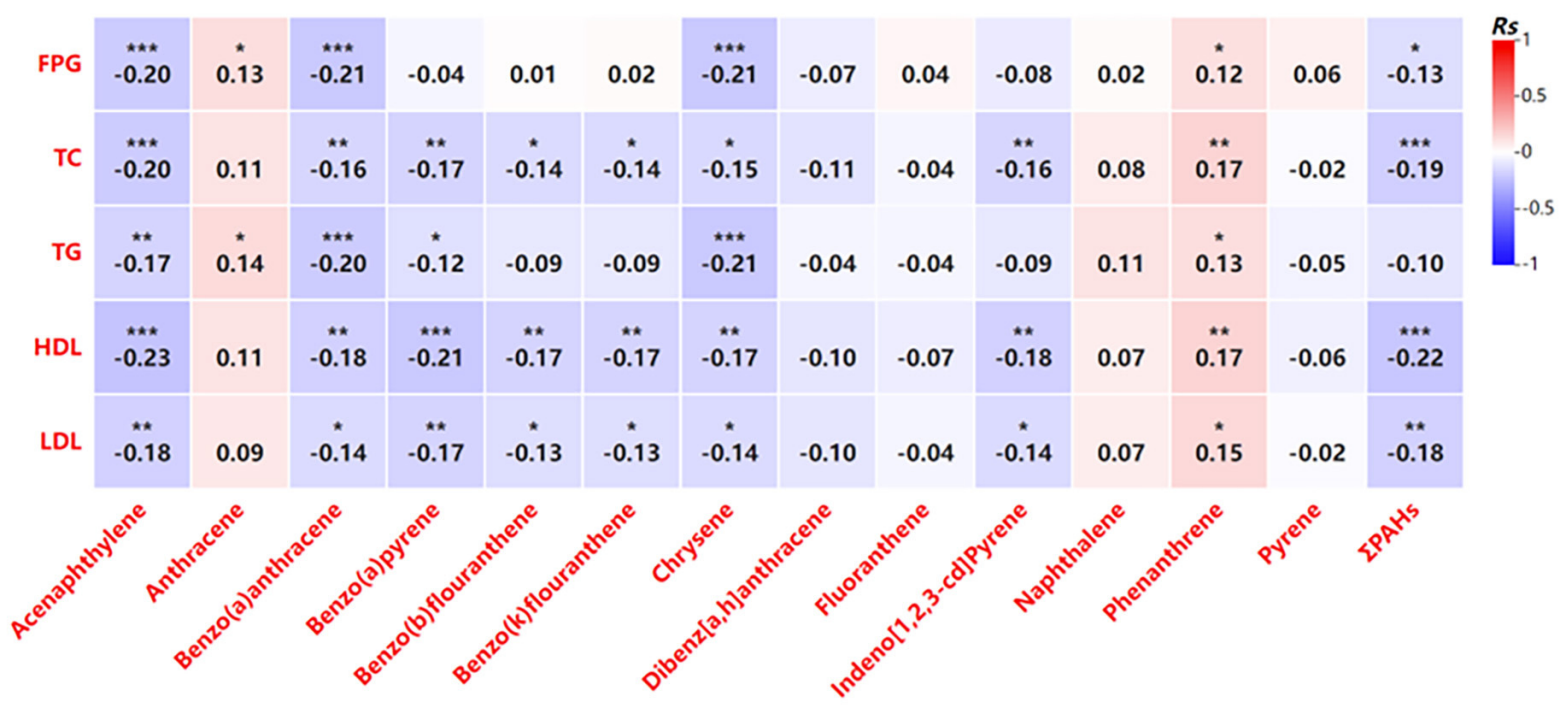
Correlation analysis of the indicators related to glycolipid metabolism PAH levels in umbilical cord blood. *P < 0.05, **P < 0.01, ***P < 0.001.

Further analysis revealed that increased PAH exposure was associated with adverse effects on glucose and lipid metabolism in pregnant women (Figure 2). Higher PAH concentrations showed negative correlations with key metabolic indicators, including total cholesterol, triglycerides, and HDL cholesterol (Figure 2). Similar trends were observed in newborns, where certain PAHs were weakly correlated with lower levels of metabolic markers such as fasting glucose and HDL cholesterol (Figure 3). However, no significant correlations were identified between glucose and lipid metabolism and 8-OHdG levels in either maternal or newborn blood samples (Table 8).

**Table 8:** Multiple linear regression analysis of 8-OHdG concentration in venous blood of pregnant women and umbilical cord blood of newborns in Haikou (*n* = 300).

**Glucose and lipid metabolism indicators**	**Venous blood 8-OHdG^a^**		**Umbilical cord blood 8-OHdG^b^**	
	**rs**	***P***	**rs**	***P***
FPG	0.005	0.948	−0.035	0.005
TC	−0.069	0.404	−0.059	−0.069
TG	−0.147	0.075	−0.091	−0.147
HDL	−0.025	0.767	−0.075	−0.025
LDL	−0.024	0.777	−0.092	−0.024

^a^Correlation with 8-OHdG levels in venous blood; ^b^Correlation with 8-OHdG levels in umbilical cord blood.

## Discussion

The current study investigated PAH exposure among pregnant women and newborns in Haikou, China, with a particular focus on its potential effects on maternal and neonatal health, specifically glucose and lipid metabolism. Thirteen different PAHs were detected in both maternal venous blood and neonatal umbilical cord blood, with notable exceptions such as acenaphthene, benzo[a]pyrene, and fluorene, which were not detected in either sample. These findings underscore a significant exposure risk for pregnant women and their newborns in Haikou, driven primarily by various environmental and lifestyle factors.

Polycyclic aromatic hydrocarbons, known for their toxic properties, are introduced into the environment through various sources, including tobacco smoke, vehicle exhaust, grilled and charred foods, industrial emissions, and natural events such as forest fires and volcanic activity (22–24). The study highlighted that PAH concentrations in pregnant women's blood were influenced by factors such as proximity to major roads, cooking methods, and dietary habits during pregnancy, particularly the consumption of fruits and vegetables. High-temperature cooking methods, including grilling, roasting, and frying, were identified as significant contributors to increased PAH exposure. Traditional Chinese cooking, which often involves temperatures exceeding 200°C, can lead to the breakdown of unsaturated fatty acids in oils and foods, thereby generating PAHs (25). These findings underscore the critical role of lifestyle choices, especially dietary habits and cooking methods, in determining PAH exposure levels during pregnancy.

We also explored the relationship between PAH exposure and metabolic indicators. A weak negative correlation was observed between the concentration of certain PAHs, such as indeno[1,2,3-cd]pyrene, and fasting plasma glucose (FPG) levels in maternal blood. This finding suggests that PAHs may interact with metabolic processes in pregnant women, potentially influenced by the physiological insulin resistance characteristic of pregnancy. The study further hypothesized that elevated estrogen levels during pregnancy might play a protective role in regulating glucose metabolism. As estrogen levels increase, particularly after 20 weeks of gestation, they enhance the function of pancreatic beta cells, promoting glucose uptake and utilization, which could contribute to lower blood glucose levels (26, 27). PAHs, known for their estrogen-mimicking properties, may contribute to the observed effects, although the specific mechanisms remain unclear and warrant further investigation. While the study identified a correlation between PAHs and glucose metabolism, no significant relationship was found between glucose-lipid metabolism and oxidative stress markers such as 8-OHdG, a biomarker for oxidative DNA damage (28). Although similar levels of 8-OHdG were observed in both maternal and neonatal blood, this only indicates comparable quantities of oxidative damage markers. It does not imply that the oxidative stress or damage levels are identical in mothers and infants. Neonates, as a vulnerable group, have underdeveloped systems, including the nervous and immune systems, making them more susceptible to oxidative damage. The same level of oxidative damage that might be tolerable in adults could have more significant, prolonged, or even irreversible effects on fetuses or newborns, potentially leading to lifelong consequences. These findings suggest that while PAHs may influence glucose regulation during pregnancy, their impact on oxidative stress and lipid metabolism is more complex and may involve other factors not addressed in this study. The authors hypothesized that PAHs might act synergistically with estrogen during pregnancy, but further research is needed to clarify the mechanisms by which PAHs interact with metabolic pathways in pregnant women.

The long-term implications of PAH exposure are concerning. The study highlighted that chronic exposure to low levels of PAHs, even among non-pregnant individuals, could result in a range of health issues, including weakened immune function, respiratory disorders, cardiovascular diseases, and cancer. Being lipophilic, PAHs are transported throughout the body via lipoproteins in the bloodstream. Their ability to enter and accumulate in the human body through multiple pathways—such as inhalation, ingestion, and skin contact—underscores significant public health concerns (29, 30). PAHs can cross the placental barrier, and the study found a correlation between PAH concentrations in maternal blood and cord blood, suggesting that these toxic compounds may affect fetal development, particularly lipid metabolism. Notably, the detection rate of some PAHs in newborn umbilical cord blood was even higher than in maternal venous blood. Since newborns are not directly exposed to PAHs, the compounds detected in their blood are derived from the mother. However, some PAHs in the maternal body may be metabolized into different forms, and various types of PAHs have different abilities to cross the placenta. If a metabolized PAH has a particularly strong ability to penetrate the placental barrier, it could result in higher concentrations of that specific PAH in the umbilical cord blood compared to maternal venous blood.

The research also highlighted elevated levels of total cholesterol (TC) and triglycerides (TG) in maternal blood samples, which is typical during pregnancy as the body prepares for increased energy demands and supports fetal growth (31). These elevated lipid levels, combined with PAH exposure, may have long-term effects on both maternal and fetal cardiovascular health (32, 33). However, the study acknowledges several limitations. It was conducted solely in Haikou, limiting the generalizability of the findings to other regions of China or globally. Additionally, the sample size was relatively small, and the study lacked air quality monitoring data, which could have provided more comprehensive insights into environmental PAH exposure levels. Future studies with larger, more diverse populations, and better environmental monitoring are needed to further explore the connections between PAH exposure and health outcomes during pregnancy.

In conclusion, this study underscores the importance of addressing PAH exposure among pregnant women, given its potential impact on both maternal and fetal health. Reducing PAH exposure through lifestyle modifications, improved air quality, and public health interventions could help mitigate the risks associated with these toxic compounds. Further research is necessary to deepen the understanding of PAHs' role in metabolic regulation during pregnancy and to develop effective strategies for protecting maternal and neonatal health.

## Data Availability

The raw data supporting the conclusions of this article will be made available by the authors, without undue reservation.
