# Supplementary material for: Sources of polycyclic aromatic hydrocarbons exposure and their effects on glycolipid metabolism in pregnant women and their newborn in Haikou City, China
Source: Front Public Health. 2025 Jan 20;12:1510517. doi: 10.3389/fpubh.2024.1510517 (PMC11788389; doi:10.3389/fpubh.2024.1510517)
Supplement: Supplementary file 1 [file Table_1.docx]

**Table S1 Parameters for GC-MS detection of the 16 PAHs**

| **Compound** | **Molecular weight** | **Parent ion** | **Product ion** | **Secondary product ion** | **Collision energy** | **Residence time** | **Retention time** | ***R^2^*** | **Detection limitation** |
| --- | --- | --- | --- | --- | --- | --- | --- | --- | --- |
| **Naphthalene** | 128.18 | 128.1 | 127 | 101 | 19 | 100 | 5.41 | 0.9912 | 0.13 |
| **Acenaphthylene** | 152.2 | 151.1 | 151 | 150 | 30 | 150 | 7.58 | 0.9939 | 0.43 |
| **Acenaphthene** | 154.2 | 154.2 | 152 | 151 | 33 | 100 | 7.69 | 0.9903 | 0.12 |
| **Fluorene** | 166.23 | 165.2 | 165 | 164 | 30 | 150 | 7.82 | 0.9912 | 0.02 |
| **Phenanthrene** | 178.24 | 177.3 | 176 | 152 | 23 | 100 | 8.79 | 0.9912 | 0.03 |
| **Anthracene** | 178.24 | 178.2 | 176 | 153 | 23 | 150 | 11.42 | 0.9912 | 0.05 |
| **Fluoranthene** | 202.26 | 202.2 | 201 | 200 | 23 | 100 | 11.59 | 0.9937 | 0.02 |
| **Pyrene** | 202.26 | 201.2 | 201 | 200 | 23 | 160 | 15.93 | 0.9937 | 0.03 |
| **Benzo(a)pyrene** | 252.3 | 252.1 | 251 | 202 | 37 | 100 | 16.61 | 0.9988 | 0.42 |
| **Chrysene** | 228.3 | 228.0 | 250 | 202 | 35 | 150 | 20.15 | 0.995 | 0.04 |
| **Benzo[g,h,i]perylene** | 76.3 | 276.0 | 276 | — | 40 | 150 | 20.24 | 0.9958 | 0.21 |
| **Benzo(a)anthracene** | 228.3 | 228.1 | 274 | 271 | 30 | 150 | 22.79 | 0.9948 | 0.02 |
| **Benzo(b)flouranthene** | 252.3 | 224.2 | 222 | 220 | 37 | 100 | 23.42 | 0.9987 | 0.11 |
| **Benzo(k)flouranthene** | 252.3 | 252.1 | 250 | 249 | 37 | 150 | 25.93 | 0.9977 | 0.31 |
| **Indeno(1,2,3-cd)pyrene** | 276.3 | 275.2 | 275 | 272 | 43 | 80 | 26.15 | 0.9958 | 0.20 |
| **Dibenz(a,h)anthracene** | 278.3 | 278.0 | 276 | — | 43 | 80 | 26.58 | 0.9949 | 0.02 |
